# Recent Advanced Technologies for the Characterization of Xenobiotic-Degrading Microorganisms and Microbial Communities

**DOI:** 10.3389/fbioe.2021.632059

**Published:** 2021-02-10

**Authors:** Sandhya Mishra, Ziqiu Lin, Shimei Pang, Wenping Zhang, Pankaj Bhatt, Shaohua Chen

**Affiliations:** ^1^State Key Laboratory for Conservation and Utilization of Subtropical Agro-Bioresources, Guangdong Province Key Laboratory of Microbial Signals and Disease Control, Integrative Microbiology Research Centre, South China Agricultural University, Guangzhou, China; ^2^Guangdong Laboratory for Lingnan Modern Agriculture, Guangzhou, China

**Keywords:** bioremediation, microorganisms, xenobiotics, omics, bioinformatics

## Abstract

Global environmental contamination with a complex mixture of xenobiotics has become a major environmental issue worldwide. Many xenobiotic compounds severely impact the environment due to their high toxicity, prolonged persistence, and limited biodegradability. Microbial-assisted degradation of xenobiotic compounds is considered to be the most effective and beneficial approach. Microorganisms have remarkable catabolic potential, with genes, enzymes, and degradation pathways implicated in the process of biodegradation. A number of microbes, including *Alcaligenes, Cellulosimicrobium, Microbacterium, Micrococcus, Methanospirillum, Aeromonas, Sphingobium, Flavobacterium, Rhodococcus, Aspergillus, Penecillium, Trichoderma, Streptomyces, Rhodotorula, Candida*, and *Aureobasidium*, have been isolated and characterized, and have shown exceptional biodegradation potential for a variety of xenobiotic contaminants from soil/water environments. Microorganisms potentially utilize xenobiotic contaminants as carbon or nitrogen sources to sustain their growth and metabolic activities. Diverse microbial populations survive in harsh contaminated environments, exhibiting a significant biodegradation potential to degrade and transform pollutants. However, the study of such microbial populations requires a more advanced and multifaceted approach. Currently, multiple advanced approaches, including metagenomics, proteomics, transcriptomics, and metabolomics, are successfully employed for the characterization of pollutant-degrading microorganisms, their metabolic machinery, novel proteins, and catabolic genes involved in the degradation process. These technologies are highly sophisticated, and efficient for obtaining information about the genetic diversity and community structures of microorganisms. Advanced molecular technologies used for the characterization of complex microbial communities give an in-depth understanding of their structural and functional aspects, and help to resolve issues related to the biodegradation potential of microorganisms. This review article discusses the biodegradation potential of microorganisms and provides insights into recent advances and omics approaches employed for the specific characterization of xenobiotic-degrading microorganisms from contaminated environments.

## Introduction

The environment is everything that naturally surrounds us and affects our daily lives on Earth. A safe and healthy environment is essential for the existence of life on this planet. However, in the era of advanced industrialization and urbanization, various anthropogenic activities are largely responsible for the introduction of toxic and hazardous pollutants such as environmental xenobiotics (Embrandiri et al., 2016; Malla et al., 2018; Bhatt et al., 2020a; Rodriguez et al., 2020). Xenobiotics are chemical substances not naturally produced or expected to be present within organisms. The term “xenobiotic” is usually used in the context of environmental pollutants to refer to synthetic compounds produced in large volumes for industrial, agricultural, and domestic use (Embrandiri et al., 2016; Atashgahi et al., 2018; Dinka, 2018). There is growing public concern over the wide range of xenobiotic compounds being introduced, deliberately or accidentally, into the environment, which involves high potential risk to humans and animals (Jacob and Cherian, 2013; Hashmi et al., 2017; Zhu et al., 2017; Dinka, 2018). Environmental xenobiotics include pesticides, polycyclic aromatic hydrocarbons (PAHs), pharmaceutical active compounds (PhACs), personal-care products (PCPs), phenolics, chlorinated compounds, and other industrial chemicals. Their increasing frequency in different environmental compartments has raised concerns about their potential adverse effects (Crinnion, 2010; Kim et al., 2013; Embrandiri et al., 2016; Tsaboula et al., 2016; Dhakal et al., 2017). Their toxicity results in unprecedented health hazards and risks to environmental safety and security (Godheja et al., 2016; Dovrak et al., 2017; Burgos-Aceves et al., 2018; Ravindra and Haq, 2019). Once xenobiotics are released into the environment, they can bioaccumulate within the food chain due to their high affinity toward organic substances, and produce toxic adverse effects toward natural ecosystems, humans, and animals (Pedersen et al., 2003; Iovdijova and Bencko, 2010; Maurya, 2016). Consequently, they can cause severe chronic effects such as respiratory tract infections, damage to the immune system, pulmonary bronchitis, dysfunction of the nervous system, disruption of the endocrine system, behavioral and developmental disorders, and carcinogenic and mutagenic effects (Sajid et al., 2015; Zhu et al., 2017; Dinka, 2018; Catron et al., 2019; Mishra et al., 2019; Bertotto et al., 2020). Thus, xenobiotic contamination represents a persistent anthropogenic threat and raises serious environmental concerns. Various physical and chemical treatment methods such as coagulation, filtration, adsorption, chemical precipitation, electrolysis ozonation, etc. have been used for the degradation and detoxification of such xenobiotic compounds, but not all these methods are very useful due to their high cost, waste-disposal problem and generation of toxic by-products that are sometimes more hazardous than the parent compound. In contrast, the biological remediation method, “bioremediation,” is a widely accepted clean-up strategy for the degradation of xenobiotics from contaminated environments without producing harmful products (Paul et al., 2005; Perelo, 2010). Bioremediation involves the metabolic capabilities of microorganisms in the removal of pollutants and thus, is the most suitable and promising technology these days (Gilliespie and Philp, 2013; Azubuike et al., 2016).

Microbial remediation of xenobiotic compounds is regarded as a superficial, proficient, economically feasible approach that uses a wide range of microorganisms to consume organic pollutants as carbon or nitrogen supplements to sustain their developmental activities (Chen et al., 2013; Mahmoud, 2016; Arora et al., 2018; Ortiz-Hernandez et al., 2018; Siles and Margesin, 2018; Zhan et al., 2018; Bhatt et al., 2020b). Microorganisms are ubiquitous in nature, and diverse microbial communities thrive in natural and extreme stress environments, including soil, water, the human gut, hydrothermal vents, acid mine runoff, and oil reservoirs (Cycoń and Piotrowska-Seget, 2016; Jalowiecki et al., 2016; Ding et al., 2017; Aguinga et al., 2018; Wang Y. F. et al., 2018; Zierer et al., 2018; Delegan et al., 2019; Arora, 2020; Shekhar et al., 2020). Microbial populations exhibit potential for the remediation of any contaminated environment because of their genetic diversity and functionality (Chen et al., 2015; Bastida et al., 2016; Bhatt and Barh, 2018; Dangi et al., 2018). Therefore, the study of microbial population existing in contaminated environments provides a significant knowledge of specific microbial characteristics that improve degradation rates. However, effective implementation of microbial remediation strategies needs advanced technical approaches, which provide an in-depth understanding about the dynamics aspects of microbial activity and survival under stressed environment (Rastogi and Sani, 2011; Lima-Morales et al., 2016; Mao et al., 2019). The development in molecular, biotechnological, bioinformatics and system biology tools pertaining to bio-remedial problems have provided gene level mechanisms of bioremediation (Ahmad and Ahmad, 2014; Aora and Bar, 2014; Singh D. P. et al., 2018; Jaiswal and Shukla, 2020; Nkongolo and Kotha, 2020; Wolf et al., 2020). Moreover, the direct study of microorganisms in a contaminated environment including the whole microbial population granted a new frontier of the scientific community to share the knowledge of the uncultured microbial world (Zepeda et al., 2015; Zhao Q. et al., 2017; Panigrahi et al., 2019; Yan et al., 2020). The development of advance molecular tools and a better understanding of microbial metabolic and genetic structures and functions have accelerated encroachment in recombinant engineering techniques to enhanced bioremediation for removal of environmental pollutants (Ram et al., 2005; Temperton and Giovannoni, 2012; Singh V. et al., 2018; Stein et al., 2018; Delegan et al., 2019; Marco and Abram, 2019; Puckett et al., 2020). Soil is the most dynamic environment for the enormous microbial population of immense diversity. It has been estimated that one gram of soil approximately contains 10^9^ bacterial cells, but only <1% of these may be culturable in the laboratory (Rossello-Mora and Amann, 2001). Culture based identification of diverse microbial population in a contaminated environment, is a challenging task, which is limited to fast-growing microbial diversity (Gillbride et al., 2006). Thus, modern culture-independent molecular techniques represent a feasible approach to unrevealing the diversity and functional dynamics of microbial population in contaminated environments. Moreover, the advanced innovation in molecular tools and techniques provides new insights and changes the traditional research trend in the field of bioremediation (Malik et al., 2008; Shah et al., 2011; Devarapalli and Kumavnath, 2015; Mahmoud, 2016; Biswas and Sarkar, 2018; Shakya et al., 2019). Omics technologies are the result of advanced molecular techniques, which involved direct characterization of genome structure of microorganisms, devoid of their culture sample (Segata et al., 2013; Biswas and Sarkar, 2018; Jaiswal et al., 2019; Yu K. et al., 2019). Therefore, the applications of modern molecular techniques like metagenomic, transcriptomic, proteomic generates relevant information on genes and proteins expression levels in whole microbial communities under contaminated environments attempted to unravel the mechanism of microbial degradation and successful execution of bioremediation (Keller and Hettich, 2009; Yang, 2013; Malla et al., 2018; Bharagava et al., 2019; Marco and Abram, 2019; Rodriguez et al., 2020). These methods are comparatively efficient, quicker, and accurate, which overcome the limitations of conventional molecular techniques. It explores the advanced microbial degradation mechanism of xenobiotics, their metabolic activities, genetic regulation and molecular-biology aspects (Cycoń et al., 2017; Gutierrez et al., 2018; Gutleben et al., 2018; Mishra et al., 2020). Hence, this review article highlights the biodegradation potential of microorganisms and provides insights into recent advance methods of “omics” technologies employed in microbial degradation and remediation purpose of xenobiotics and their perspectives in modern biological research.

## Bioremediation Potential of Microorganisms for Xenobiotic Compounds

**Figure 1:**
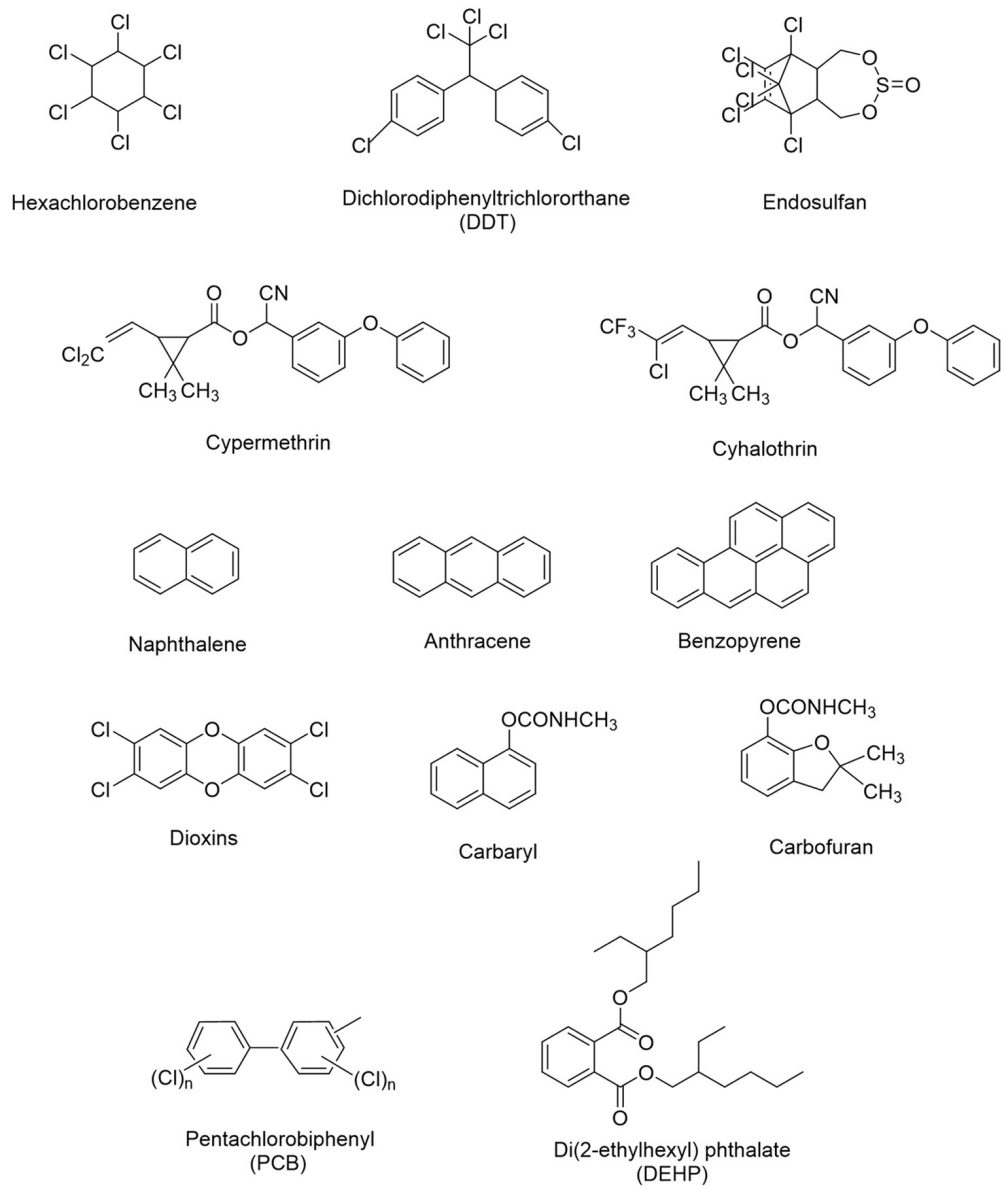
Chemical structures of several xenobiotic compounds.

The application of microorganisms in removing xenobiotics from soil, water or sediments through complete transformation or mineralization into harmless end products like CO_2_ and H_2_O is a basic concept of bioremediation strategy (Ortiz et al., 2013; Singh et al., 2016). Different microorganisms including bacteria (*Pseudomonas, Alcaligenes, Cellulosimicrobium, Microbacterium, Micrococcus, Methanospirillum, Aeromonas, Bacillus, Sphingobium, Flavobacterium*, and *Rhodococcus*), fungi (*Aspergillus, Penecillium, Trichoderma*, and *Fusarium*), and yeasts (*Pichia, Rhodotorula, Candida, Aureobasidium*, and *Exophiala*) have been reported to be involved in the efficient biodegradation of xenobiotic compounds from contaminated soil/water environments, due to their exceptional bioremediation potential (Sathishkumar et al., 2008; Nzila, 2013; Sunita et al., 2013; Zhao Q. et al., 2017; Bharadwaj, 2018; Yang J. et al., 2018; Yang T. et al., 2018; Yu Y. et al., 2019; Bhatt et al., 2020c). The biodegradation ability of microbes is greatly influenced by interactive ecological factors including soil, salinity, temperature, carbon source, moisture content, pH, nitrogen sources, inoculums concentration, etc. (Megharaj and Naidu, 2010; Wu et al., 2014; Bhatt et al., 2019). Microorganisms harbor remarkable catabolic potential, genes, enzymes, and degradation pathways implicated in the process of bioremediation, which might be responsible in the evolution of novel traits and characters (Widada et al., 2002; Scholer et al., 2017; Yan et al., 2018; Zhu et al., 2020). Moreover, microbial plasmids believed to be responsible for the continuous progression, evolution, and distribution of novel biodegradable genes/enzymes (Zhang et al., 2016; Jeffries et al., 2019). These novel genes/enzymes have endowed microorganism's biodegradation capability to remove or detoxify a wide variety of environmental pollutants due to their inheritance horizontal gene transfer property (Singh V. et al., 2018; Jaiswal et al., 2019; Li et al., 2019; Phale et al., 2019; French et al., 2020). The microbial remediation process can be further improved via successful application of genome editing and biochemical techniques that modify existing strains and result in the development of a genetically modified organism capable of simultaneously degrading several xenobiotics (Shanker et al., 2011; Zhang et al., 2016; Hussain et al., 2018; Janssen and Stucki, 2020). The advancement of genetic manipulation technology gives more clear information and explores future prospects of bioremediation of xenobiotics through highly proficient microorganisms (Sayler and Ripp, 2000; Shapiro et al., 2018; Wong, 2018; Liu et al., 2019). The chemical structures of several xenobiotic compounds are presented in Figure 1.

**Figure 2:**
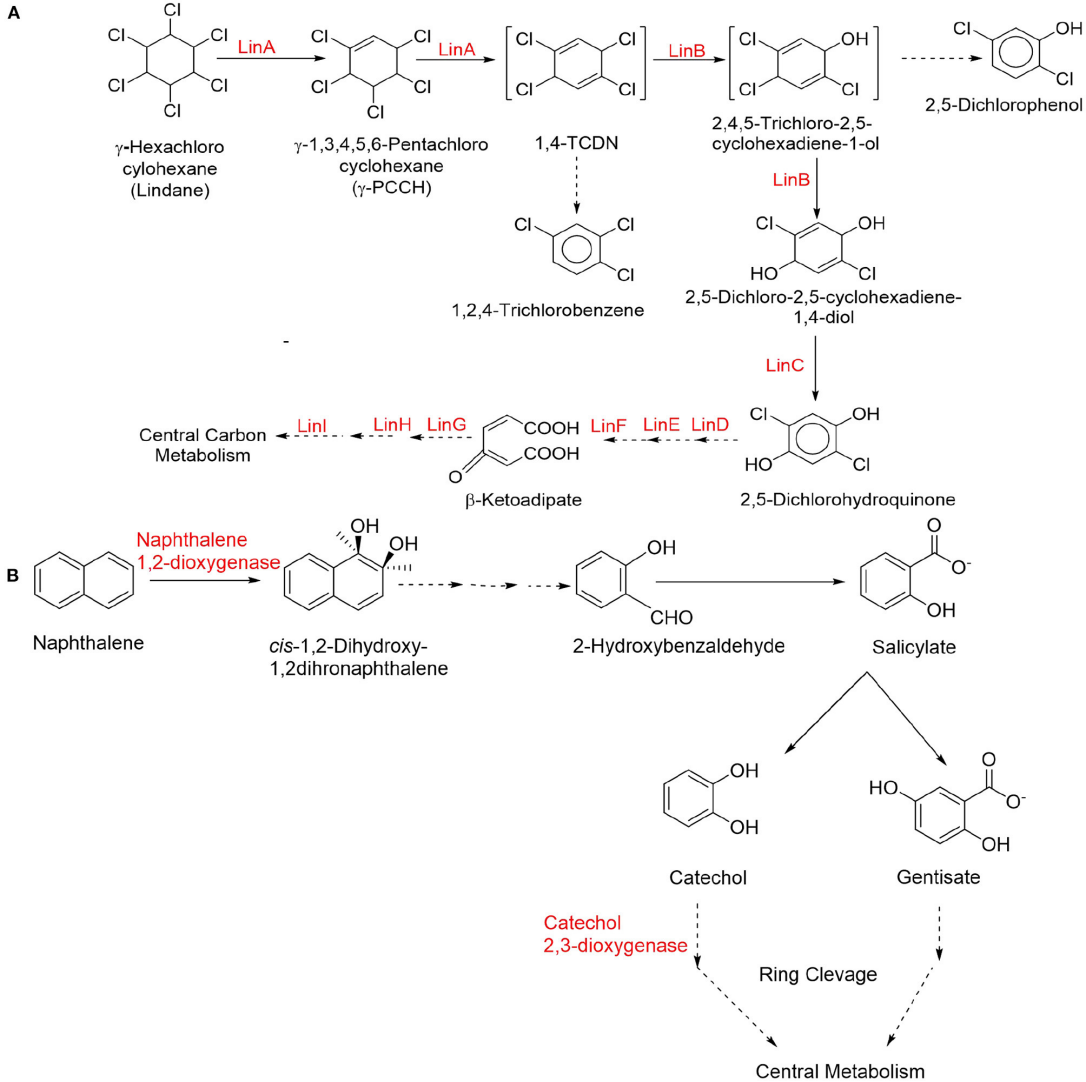
Simplified catabolic degradation pathways of organochlorine and polycyclic aromatic hydrocarbon (PAH). Compounds **(A)** lindane (organochlorine) (Endo et al., 2005; Geueke et al., 2013) and **(B)** naphthalene (PAH) (Kiyohara et al., 1994; Dutta et al., 1998; Bamforth and Singleton, 2005; Baboshin et al., 2008).

Synthetic pesticides are the major example of xenobiotics especially the organochlorine pesticides (OCPs) that used extensively worldwide for a long period of time in agriculture as well as in insect control program. Several OCPs such as aldrin, dieldrin, dichloro diphenyl trichloro ethane (DDT), benzene hexachloride (BHC), and hexachlorocyclohexane are highly toxic in nature due to their stability and bioaccumulative property (Aktar et al., 2009; Jayaraj et al., 2016; Awasthi and Awasthi, 2019; Pang et al., 2020). Lindane (γ-hexachlorocyclohexane) is one of the highly toxic organochlorine xenobiotic compound, which is well-studied for its microbial biodegradation through physical (soil structure, carbon and oxygen gradients, pH, and temperature) and chemical (dechlorination, dehydroxylation, and dehydrogenation) interactions (Kaur and Kaur, 2016; Bashir et al., 2018; Zhang et al., 2021). The increasing concentration of lindane residues into the environment imposes severe health hazards such as carcinogenicity, mutagenicity, endocrine disruption, and immune-suppression diseases into the humans and other organisms (Cuozzo et al., 2017; Zhang et al., 2020). Bacterial strains genera such as *Bacillus, Burkholderia, Pseudomonas, Kocuria, Archromobacter, Sphingomonas, Chromohalobacter*, demonstrated lindane biodegradation under axenic as well as anoxic conditions via dehydrogenation, dehydrochlorination, and hydroxylation, results in complete degradation or mineralization (Giri et al., 2014; Cuozzo et al., 2017; Wang W. et al., 2018; Nagata et al., 2019; Zhang et al., 2020). A simplified catabolic pathway of lindane is presented in Figure 2.

Pyrethroids are broad spectrum pesticide mainly used against agricultural and household pests. Cypermethrin, cyhalothrin, deltamethrin, cyfluthrin, bifenthrin are the common example of synthetic pyrethroids (Chen et al., 2012, 2014; Bhatt et al., 2019; Zhan et al., 2020). These pesticides are highly toxic and persistent and can cause molecular toxicity, neurotoxicity, and reproductive toxicity (Sharma et al., 2018; Bhatt et al., 2019; Gammon et al., 2019). One of the pyrethroid i.e., cypermethrin can cross the blood-brain barrier and induces neurotoxicity and motor deficits (Singh et al., 2012). The persistence of these pesticides in the environment poses a severe threat to humans and other non-target terrestrial and aquatic organisms (Burns and Pastoor, 2018; Ullah et al., 2018; Lu et al., 2019). Microbial strains such as *Acinetobacter, Trichoderma, Roultella, Pseudomonas, Cunninghamella*, and *Bacillus* have been reported for their efficient degradation of broad spectrum pesticides like cypermethrin and other pyrethroid pesticides through pyrethroid hydrolases (Cycoń and Piotrowska-Seget, 2016; Zhan et al., 2018; Bhatt et al., 2019; Chen and Zhan, 2019). Co-metabolism exhibiting strains include *Flavobacterium, Sphingomonas, Arthrobacter, Azotobacter, Achromobacter, Microbacterium, Brevibacterium, Rhodococcus, Trichoderma*, and *Aspergillus* demonstrated their pollutant degradation capability, exclusive of pollutant consumption as an energy resource (Nzila, 2013).

Polychlorinated bis-phenyles (PCBs) are classified as persistent organic pollutants with high toxicity (Lallas, 2001; Pathiraja et al., 2019). They have been linked to chronic effects in humans including immune system damage, decreased pulmonary function, bronchitis, and interference with hormones leading to cancer (Schecter et al., 2006). Microbial degradation of polychlorinated bis-phenyles (PCBs) is a promising remediation technology involving two major pathways: aerobic degradation and anaerobic dehalogination (Abraham et al., 2002; Pathiraja et al., 2019). Bacterial strains *Pseudomonas, Rhosocossus, Comamonas, Burkholderia*, and *Bacillus* have been characterized for the oxidative degradation of PCBs through ring cleavage resulting into a common by product of chlorbenzoic acid (Anyasi and Atagana, 2011; Jing et al., 2018).

Polycyclic aromatic hydrocarbons (PAHs) are potent environmental contaminants and xenobiotics that are widely distributed in the environment due to the incomplete combustion of organic matter. PAHs have moderate to high acute toxicity to aquatic life and birds (Abdel-Shafy and Mansour, 2016; Pandey et al., 2017). Some PAHs such as Anthracene, benzo(a)pyrene, phenanthrene, and naphthalene are well-known to produce harmful biological effects such as genotoxicity, mutagenicity, and carcinogenicity and therefore pose a serious threat to the human health (Kim et al., 2013; Lin et al., 2020). Microorganisms belonging to *Sphingomonas, Sphingobium*, and *Novosphingobium* have been found as efficient degrader of PAHs (Lee et al., 2016b; Fida et al., 2017; Auti et al., 2019). *Rhodococcus, Cunninghamella, Pleurotus ostreatus, Oscillatoria, Agmenellum quadriplicatum, Brevibacterium*, and *Nocardiodes* have been widely shown to metabolize particularly naphthalene and phenanthrene (Ghosal et al., 2016; Siles and Margesin, 2018). Ortega-Gonzalez et al. (2015) demonstrated that *Amycolaptosis* sp. Poz14 degraded 100% of naphthalene and 37.87% of anthracene within 45 days. A PAH-degrading marine bacterium *Cycloclasticus* sp. has been isolated from sea sediments capable to breakdown xenobiotic naphthalene, pyrene, phenanthrene, and other aromatic hydrocarbons into their supplementary products through enzymatic pathways (Wang W. et al., 2018). A simplified catabolic pathway of naphthalene is presented in Figure 2.

Phthalates or esters of phthalic acids are synthetic xenobiotic chemicals, which are extensively used as plasticizers added to polyvinyl chloride to improve its flexibility and hardness (Crinnion, 2010; Singh and Li, 2011; Przybylinska and Wyszkowski, 2016). Phthalates are readily released into the environment and create a risk of exposure for humans and other living organisms due their endocrine disrupting behavior (Przybylinska and Wyszkowski, 2016). They cause infertility, reproductive and developmental toxicity in humans and animals (Singh and Li, 2011; Przybylinska and Wyszkowski, 2016). Aerobic and anaerobic microbial degradation of xenobiotic phthalic acid, and isophthalaic acid considered as the most effective means of their removal from the environment (Junghare et al., 2019; Boll et al., 2020). Di-2(ethyehxyl) phthalate (DEHP) is the most common member of phthalates, which are extensively used as plasticizer in plastics and disposable medical materials (Singh and Li, 2011). DEHP is best known as an endocrine disrupter and can produce neural, heptotoxic, cardiotoxic, and carcinogenic effects on humans and animals (Rowdhwal and Chen, 2018). *Arthrobacter, Pseudomonas, Gordonia, Providencia, Acinetobacter, Microbacterium*, and *Rhodococuus* have been identified as efficient DEHP degrading bacteria (Nahurira et al., 2017; Yang T. et al., 2018).

In addition, the microbial consortium gained greater attention in bioremediation rather than pure microbial monocultures. The consortia cultures are better equipped in terms of metabolic and pollutant removal capability owing to their constant revelation of contaminant and promising mutual relationship with other available strains (Patowary et al., 2016). Interestingly, microbial consortia can alleviate the metabolic limitations of single microbial culture and enhance biodegradation process by their miscellaneous assemblage of bacterial populations employed with extensive degradation potential (Zafra et al., 2016; Li et al., 2020).

## Recent Advanced Technologies Employed in Bioremediation for Identification and Characterization of Microorganisms and Microbial Communities

**Figure 3:**
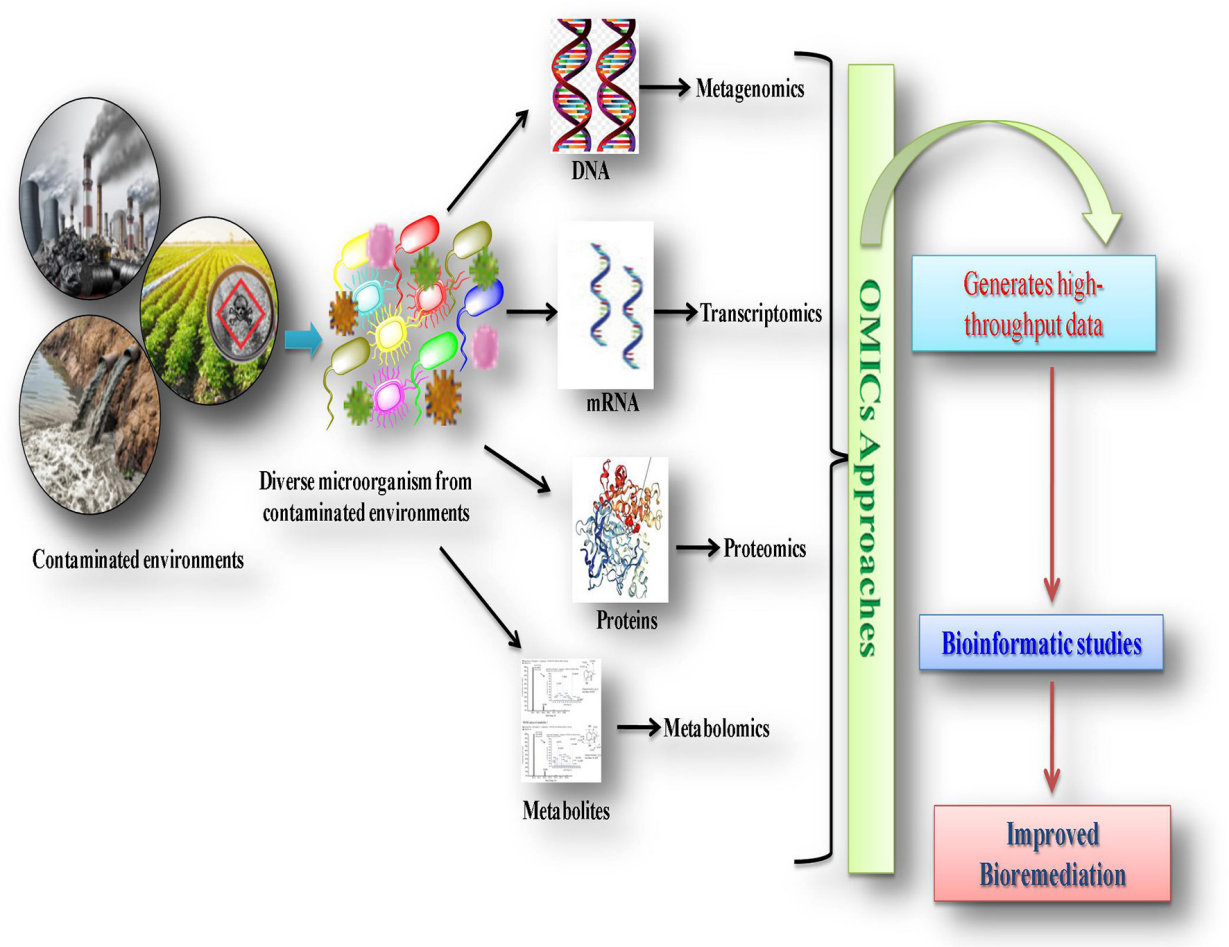
Graphical presentation of integrated approach of advanced technologies in biodegradation of xenobiotic compounds.

There are several revolutionary advanced molecular practices, including genomics, metagenomics, proteomics, transcriptomics, and metabolomics, which deliver deeper insights into microbial activities with respect to their genes, proteins, mRNA expression levels, enzymes and metabolic pathways with changing environments (Figure 1). The integrated approach of these multiple technologies in the field of bioremediation is termed as the “omics approach,” used for the undeviating characterization of biological macromolecules, and their specific genetic and molecular structures and function mechanisms in a set of microorganisms/microbial communities (Desai et al., 2009; Yang, 2013; Godheja et al., 2014; Franzosa et al., 2015; Chandran et al., 2020). The application of omics technologies provides comprehensive insights into microbial populations, their mechanisms of interaction with pollutants, metabolic activities, and genetic-regulation and molecular-biology aspects (Akinsanya et al., 2015; Kaul et al., 2016; Misra et al., 2018; Marco and Abram, 2019; Huang et al., 2021). Moreover, these approaches can broaden our knowledge of the so-called “viable but non-culturable (VBNC)” bacteria and their potentially novel pathways for degrading environmental pollutants (Oliver, 2010; Bodor et al., 2020). It is believed that these uncultured bacteria may play an important role in the biodegradation of environmental pollutant. However, little is known about the VBNC bacteria as these bacteria cannot be cultivated on conventional media and are very different from cells (Su et al., 2013). VBNC cells exhibit metabolic and respiratory activities and may perform transcription and gene expression, which allows them to recover culturability (Oliver et al., 2005; Zhao X. et al., 2017). The VBNC bacteria could be resuscitated in favorable conditions by an autoinducer (AI-2) (Bari et al., 2013) or resuscitated promoting factor (Rpf) (Li et al., 2015). The VBNC bacterial cells are detected on the basis of their viability. Several culture-independent molecular-based methods such as denaturing and temperature gradient gel electrophoresis (DGGE/TGGE), fluorescent *in situ* hybridization (FISH), terminal restriction fragment length polymorphism (T-RFLP), fatty acid methyl ester (FAME), and next-generation sequencing (NGS) technology are used for obtaining important information about the structural composition and genetic diversity of unculturable microorganisms (Zhao X. et al., 2017; Bodor et al., 2020). Selective gene amplification is an emerging approach to detect viable cells. Zhong et al. (2016) developed a real-time fluorescence LAMP technique combined with PMA (propidium monoazide), a high-affinity photolysis DNA nucleic acid dye applied for the detection of VBNC *V. parahaemolyticus*. Thus, the combined use of these advanced molecular technologies with bioinformatic approaches increases understanding and brings in a new era of unrevealed soil microbial communities, as well as their associated mechanisms of biodegradation for their future applications in bioremediation (Figure 3; Mocalli and Benedetti, 2010; Kumar et al., 2016; Dangi et al., 2018; Pandey et al., 2019; Pinu et al., 2019).

### Genomics and Metagenomics

Genomics and metagenomics are powerful tools for analyzing microbial communities at the genomic level from various contaminated environments. This technology gives a new array to environmental microbiologists for understanding unculturable microbiota with a genetic variability of microbial communities (Devarapalli and Kumavnath, 2015; Zhu et al., 2018; Awasthi et al., 2020). It gives more details about the particular degradation potential of microbial communities, as it directly entails the whole-genome sequence from environmental samples (Table 1). Metagenomic studies unblock traditional ways of uncultured microorganisms and explore their genetic advantage in the process of bioremediation (Rahimi et al., 2018; Nascimento et al., 2020). Complete genome-sequence data of some important microbial strains, including *Pseudomonas aeruginosa* KT2440, *Shewanella oneidensis* MR-1, *Deinococcus indicus* R1, and *Dehalococcoides mccartyi* WBC-2 have already been given, which is pertinent to successful bioremediation (http://www.tigr.org). The new genes can tell too much about the degradation capability and substrate specificity.

**Table 1:** Microorganisms and microbial communities using genomic and metagenomic approaches in biodegradation.

**S. no**.	**Microorganisms/ microbial communities**	**Isolation source**	**Xenobiotics/ pollutants**	**Comments/result**	**References**
1.	*Sphingomonas and Sphingobium*	Soil	Aromatic hydrocarbons	Whole-genome sequence gives insights into the presence of *bph* and *xyl* gene clusters in six bacterial strains for degradation of aromatic hydrocarbon and other xenobiotics.	Zhao Q. et al., 2017
2.	*Gordonia* sp. 1D	Oil-refinery soil	Naphthalene	Genomic analysis of *Gordonia* sp. 1D showed that it contains gene clusters of alkaline hydroxylase, genes of dibenzothiophene and naphthalene metabolism intermediates, which are involved in the degradation of naphthalene and other environmental pollutants.	Delegan et al., 2019
3.	*Bacillus megaterium* STB1	Contaminated soil	Xenobiotics	Genomic analysis of *Bacillus megaterium* STB1 demonstrated that it contains genes responsible for degradation of xenobiotics and developing stress-resistance mechanisms.	Nascimento et al., 2020
4.	*Cycloclasticus sp*. P1	Deep sea sediments	Polycyclic aromatic hydrocarbons (PAHs)	Genomic analysis of strain P1 revealed that six ring hydroxylating dioxygenase (RHD) enzyme were responsible for the degradation of PAH compounds like pyrene and phenanthrene.	Wang W. et al., 2018
5.	Microbial community	Hydrocarbon contaminated soil	Petroleum hydrocarbon	Gammaproteobacteria and Bacteroid classes within bacterial communities were found to be actively involved in total petroleum hydrocarbon (TPH) degradation.	Siles and Margesin, 2018
6.	Microbial community	Sugarcane farms, Australia	Organophosphates	Functional metagenomics explores the capability of different microbial profiles to predict the degradation of organophosphates in different soil types.	Jeffries et al., 2019
7.	Microbial communities	River sediments		Metagenomic study explored the correlation between diverse microbial communities and environmental factors, and their resistance mechanism under stress environments.	Yan et al., 2020
8.	Microbial community	Contaminated soil	Di (2-ethylhexyl) phthalate (DEHP)	Members of *Actinomycetales* seemed to be the dominant degraders of di (2-ethylhexyl) phthalate (DEHP) under aerobic conditions.	Zhu et al., 2020
9.	Mixed bacterial consortium	Polycyclic aromatic hydrocarbon (PAH)-contaminated soil	PAHs	Functional metagenomics demonstrated alteration on gene clusters, and predicted intermediary degradation pathway for the mineralization of PAH compounds.	Zafra et al., 2016
10.	Microbial community	Lindane- contaminated pond sediments	Lindane	Metagenomic analysis revealed that microbial populations such as Actinobacteria, Acidobacteria, Planctomycetes, and Proteobacteria, abundantly present in contaminated pond sediments, showed lindane-degradation capabilities.	Negi and Lal, 2017
11.	Microbial community	Jet-fuel-contaminated site	Toluene and benzene	Metagenomic analysis characterized degradation potential and metabolic pathway for toluene and benzene by members of *Geobacteraceae* and *Peptococcaceae* microbiota present in jet-fuel-contaminated site.	Hidalgo et al., 2020
12.	Microbial communities	Insecticide plant soil	Organochlorines	Metagenomic analysis revealed the response of microbial community composition and diversity to organochlorine pesticide (OCP)-contaminated sites and offered potential bioremediation strategies for the mitigation of OCP-contaminated sites.	Sun et al., 2019
13.	Microbial consortium	Contaminated soil	Biphenyl	Metagenomic analysis of a bacterial consortium revealed the metabolic role of strains belonging to the genera *Pseudomonas, Rhodococcus, Bordetella, Achromobacter*, and *Varivorex* in the bioremediation of biphenyl to benzoate and benzoate to the tricarboxylic acid cycle (TCA).	Garrido-Sanz et al., 2018

Current metagenomic practices allowed for identifying the whole-genome structure of microorganisms and specifying particular genes that are attributed to encode degradative enzymes for the mineralization of xenobiotics (Zafra et al., 2016; Zhu et al., 2020). Thus, metagenomics clearly highlights the crucial role of novel genes in connecting the entire microbial population with functional diversity and structural identity. Metagenomics involves the manufacturing of metagenomic libraries that include (I) production of the proper size of DNA fragments, and ligation of these fragments into a suitable cloning vector; (II) further recombinant vectors introduced into an appropriate bacterium cloning host; (III) clones that harbor specific characters, functions, or sequences were screened for libraries (Figure 3). Moreover, the screening of metagenomic libraries can be performed by two processes, i.e., sequence-driven analysis using high-throughput sequencing, and functional analysis using phenotypic expressions (Handelsman, 2004). However, recent sequence-based metagenome analyses (such as SOLiD system of Applied Biosystems, Roche 454 sequencing) are performed without the construction of cloned libraries (Kumar et al., 2020).

Function-driven metagenomics is a potent method for studying the functional aspect of genes. It is widely used for discovering novel genes with desired functions or exploring the sequence diversity of protein families (Taupp et al., 2011; Lam et al., 2015). A function-driven analysis involves the construction and screening of metagenomic libraries to identify novel enzymes (Chakraborthy and Das, 2016; Kumar et al., 2020). Using functional metagenomics, many novel antibiotic-resistant genes were identified from environmental sources (Ngara and Zhang, 2018). The majority of metagenome-derived hydrolytic enzymes, mainly esterases and glycoside hydrolases, have been characterized biochemically and mainly originated from functional metagenomics (Steele et al., 2009; Taupp et al., 2011). A novel functional screening method, a metagenome extract thin layer chromatography (META) system, was developed by Rabausch et al. (2013) for the rapid detection of glycosyltransferase (GT) and other flavonoid-modifying enzymes from metagenomic clone libraries. It involves the screening of 38,000 clones from two different metagenomic libraries and allowed for the identification of two novel UDP glycosyltransferase (UGT) genes. Bouhajja et al. (2017) utilized function-based screening of metagenomic libraries to explore the diversity of genes and microorganisms involved in the monooxygenase-mediated toluene degradation in a hydrocarbon-polluted sediment sample.

Metagenomic approaches offer broad and reliable microbial identification on the species and strain levels, but they are much costlier methods, and the most challenging part is data investigation, which requires all short DNA sequences to pair together to assemble the final genome structure (Bragg and Tyson, 2014). The involvement of indigenous soil microorganisms in the degradation of PAHs was undertaken by Zafra et al. (2016) through a metagenomic approach. This study demonstrated the biodegradation efficiency of microbial consortia with their degradative enzymes and metabolites generated during the remediation process. The microbial-community dynamics of refined- and crude-petroleum-contaminated soil by next-generation sequencing (NGS), and their capability to degrade hydrocarbons and plant-growth-promotion potential through *in-silico* analysis was investigated by Auti et al. (2019). In this study, 16S rRNA amplicon sequencing on the Illumina MiSeq platform and PICRUSt revealed that both types of soil contained microbial communities with excellent metabolic potential for petroleum hydrocarbon (PHC) degradation. Using KEGG orthology, the abundance of functional genes involved in hydrocarbon degradation showed the presence of 61 enzyme-encoding genes, such as alkane monooxygenase, alcohol dehydrogenase, and aldehyde dehydrogenase (Auti et al., 2019). 16S rDNA or 16S rRNA gene sequencing has led to evolutionary insights into the phylogenetic and taxonomic identification of microorganisms. The 16S rRNA gene consists of several highly conserved regions interleaved with variable regions in all microorganisms and thus is highly suited as a target gene for sequencing DNA (Fuks et al., 2018; Gursoy and Can, 2019). The bacterial 16S rRNA gene generally contains nine “hypervaiable regions” that demonstrate the considerable sequence diversity of bacterial species and can be used for species identification. The 16 S rRNA gene sequence similarity between two strains provides a simple yet robust criterion for the identification of newly isolated strains, whereas phylogenetic analyses can be used to elucidate the overall evolutionary relationship between related taxa (Johnson et al., 2019). Thus, 16S rRNA gene sequencing analysis is a highly recommended cost-effective technique for the phylogenetic resolution and taxonomic profiling of microbial communities (Auti et al., 2019).

The NGS approach has completely changed microbial-community analysis, as it provides comparative details in terms of temporal and spatial data (Hidalgo et al., 2020). There are several NGS technologies, including Illumina, Ion Torrent, SOLiD, and 454 (Caporaso et al., 2012; Liu et al., 2012; Knief, 2014; Salipante et al., 2014; Machado et al., 2019). These are high-throughput sequencing techniques of ribosomal genes that quantify community structures and functions at a higher resolution, e.g., 16S rRNA in prokaryotes, and 5S or 18S rRNA genes, or the internal-transcribe-spacer (ITS) region in eukaryotes (Luo et al., 2012). The effectiveness of such NGS technologies in analyzing microbial communities from diverse environments was elucidated, validated, and documented in many studies (Brown et al., 2013; Shokralla et al., 2014; Zhou et al., 2015; Niu et al., 2016; Scholer et al., 2017). In addition, PacBio (Pacific Biosciences) and Oxford Nanopore are highly advanced, reliable, and accurate third-generation sequencing platforms applied to microbial community analysis (Lu et al., 2016; Chandran et al., 2020). Oxford Nanopore Technologies has launched a portable MinION USB nanopore that does not rely on DNA replication and has the advantage of reading full-length molecules in real time. PacBio RS II, the first commercialized genomic sequencer, developed by Pacific Biosciences, uses single-molecule, real-time (SMRT) technology and is able to sequence single DNA molecules in real time without PCR amplification (Wagner et al., 2016; Nakano et al., 2017). The complete genome sequence of atrazine-degrading *Arthrobacter* sp. ZXY-2 (Zhao X. et al., 2017) and organophosphate-degrading *Sphingobium fuliginis* ATCC 27552 (Azam et al., 2019) was analyzed using the PacBio RSII sequencing platform to gain more insight into the genetic basis and unravel its degradation potential.

Jeffries et al. (2019) performed functional metagenomic studies in pesticide-contaminated soil to explore the degradation rates of organophosphorus xenobiotic compounds. Their study demonstrated that two distinct soil groups had different functional and taxonomic profiles, and predicted biodegradation potential in rapidly and slowly degrading soil clusters. *Burkholderia, Acidomicrobium, Koribacter*, and *Bradyrhizobium* were most abundantly present in rapidly degrading clusters, whereas *Singulisphaera, Solibacter*, and *Desulfomonile* were in slowly degrading clusters. The degradation assays of organophosphorus also suggested that slow-degradation clusters had significantly higher abundances of virulence genes and metabolic pathways for fatty acids and carbohydrates. In contrast, rapid-degradation clusters contain more abundant genes related to the transposable elements, membrane transport, and nutrient cycling of nitrogen and phosphorus enzymes potentially involved in phosphorus metabolism. Moreover, rapid-degradation soils also showed a higher abundance of genes encoding phosphodiesterase enzymes, which cleave phosphodiester bonds present in organophosphorus and play a major role in pesticide degradation. Overall, this study gives an overall framework of metagenomic approaches to predict the microbial degradation of xenobiotic organophosphorus compounds.

Metagenomic analysis of a complex community of lindane-contaminated pond sediment was conducted by Negi and Lal (2017) through comparative genomics. The results of this study revealed genomic variation present in pond sediment with degradative enzymes (hydrolases, isomerases, lyases, and oxidoreductases) involved in the biodegradation of hexachlorocyclohexane and chlorobenzene (ko00361), and other xenobiotic compounds. *Cellulomonas, Micrococcus, Nocardioides, Kribbella, Isoptericola, Clavibacter, Gutenbergia, Streptomyces, Sanguibacter*, and *Kineococcus* were found to be the most dominating genera present in the aromatic-hydrocarbon-contaminated pond sediment. Genes involved in lindane metabolization, enriched with sequences for *linA* and *linB*, were also found in the pond-sediment metagenome.

Whole-metagenome sequencing of e-waste-contaminated microbial populations was conducted by Salam and Verma (2019), who demonstrated that the functional diversity and structural composition of microorganisms significantly changes due to the detrimental impact of e-waste. Denaturing gel gradient electrophoresis (DGGE) community-profiling results revealed that bacterial groups such as *Proteobacteria, Firmicutes, Bacteroidetes*, and *Chloroflexi* were decreased. Zhu et al. (2020) explored microbial assemblage and functional genes potentially involved in upstream and downstream phthalate degradation in soil via a metagenomic approach. Results indicated that bacterial taxon Actinobacteria (*Pimelobacter, Nocardioides, Gordonia, Nocardia, Rhodococcus*, and *Mycobacterium)* was a major degrader under aerobic conditions, and bacterial taxa Proteobacteria (*Ramlibacter* and *Burkholderia*), Acidobacteria, and Bacteroidetes were involved under anaerobic conditions. The members of *Geobacteraceae* and *Peptococcaceae* microbiota present in the jet-fuel-contaminated site could be exploited for their remarkable metabolic potential for the mitigation of toluene and benzene, as exposed by metagenomic analysis (Hidalgo et al., 2020).

### Transcriptomics

Transcriptomics is a remarkable tool that addresses the division of genes transcribed in any organism known as the transcriptome. It provides functional insight links involving the genome, proteome, and cellular phenotype by studying their mRNA transcriptional profiles, directly extracted from individual microbes or microbial communities (Singh and Nagaraj, 2006; McGrath et al., 2008; Bashiardes et al., 2016). Significant changes were seen in gene-expression level and their regulation in microbial communities under stressful environments for their survival. Thus, transcriptomics and metatranscriptomics provide deep analysis of a genome wide range of differently expressed genes, either of the individual cell or the entire microbial community at a specific time (Table 2; Li et al., 2014; He et al., 2015; Shakya et al., 2019). RNA seq and DNA microarrays are significantly powerful technologies to determine the mRNA expression level of every gene (Diaz, 2004). GeoChip uses key enzymes or genes to spot various microbe-mediated mechanisms for biogeochemical cycles, resistance mechanism for heavy metals, and degradation pathways of xenobiotics (He et al., 2010; Xiong et al., 2010; Xie et al., 2011). Similarly, DNA- and RNA-SIP (Stable Isotope Probing) technologies are also valuable to uncover the microbial taxa and catabolic genes that are important for the bioremediation of polluted environments (Lueders, 2015).

**Table 2:** Microorganisms and microbial communities using transcriptomic and metatranscriptomic approaches in biodegradation.

**S. no**.	**Microorganisms/ microbial communities**	**Isolation/ culture source**	**Xenobiotics/ pollutants**	**Comments/result**	**References**
1.	*Dehalococcoides mccartyi* within trichloroethene-dechlorinating community	Dechlorinated enrichment culture	Trichloro-ethane (TCE)	Transcriptomic approach identified genes encoding for rRNA, and reductive dehalogenases *tceA* and *vcrA* as the most expressed genes for TCE-dechlorinating community, while in *D. mccartyi*, hydrogenases *hup* and *vhu* were identified.	Mao et al., 2019
2.	*Rhodococcus* sp. CS-1	Drinking-water treatment plant	Phenol	Transcriptomic analysis showed that *Rhodococcus* sp. CS-1 was capable of phenol degradation via ketoadipate pathway.	Gu et al., 2018
3.	*Rhodococcus erythropolis* D310-1	Activated-sludge sample	Chlorimuron-ethyl	RNA-seq results suggested that cyt P450 carboxylesterase and glycosyltransferase genes are key genes expressing degradation of chlorimuron-ethyl.	Cheng et al., 2018
4.	*Burkholderia zhejiangensis* CEIB S4-3	Pesticide-contaminated soil	Methyl parathione	Transcriptomic analysis of CEIB S4-3 strain showed transcriptional changes occurred in response to methyl parathion, and identified expressed genes related to its biodegradation.	Castrejon-Godinez et al., 2019
5.	*Sphingomonas haloaromaticamans* P3.	Wastewater disposal site soil	Polyphenol	Transcriptomics analysis of strain P3 revealed expression patterns of catabolic genes of *ortho*-phenyl phenol degradation pathway.	Perruchon et al., 2017
6.	*Novosphingobium* sp. LH128	Contaminated soil	Phenanthrene	Transcriptomics analysis showed remarkably higher expression of phenanthrene degradation.	Fida et al., 2017
7.	*Novosphingobium resinovorum* strain SA1	Contaminated soil	Sulphanilic acid	Transcriptomic analysis showed that the strain SA1 was capable to degrade suphanilic acid into sulfonated aromatic compounds.	Hegedus et al., 2018
8.	*Pseudomonas putida* KT2440	–	2,4,6-trinitrotoluene (TNT)	Transcriptomic analysis revealed that strain KT2440 showed a high level resistance to TNT. Significant expression level changes were observes in 65 genes. Of these 39 genes were upregulated and 26 were downregulated. Detoxification related enzymes and genes encoding nitroreductases (*pnrA, xenD*, and *acpD)* were induced in response to TNT detoxification.	Farnandez et al., 2009
*9*.	*Cycloclasticus* sp. P1	Deep sea sediments	Naphthalene/ phenanthrene/ pyrene and other aromatic hydrocarbons	Transcriptomic analysis confirms that five gene clusters were involved in biodegradation of PAH compounds in strain P1.	Wang W. et al., 2018
10.	Microbial communities	Agricultural and organic soil	Aromatic hydrocarbons	Metatranscriptomic analysis showed that agricultural soil has high expression of resistant proteins, dioxygenases, metapyrocatechases, 4-hydroxyphenylpyruvates, and ring hydroxylating dioxygenases for aromatic-hydrocarbon-degrading pathways in comparison to organic soil.	Sharma et al., 2019
11.	Microbial communities.	Wheat rhizosphere	Aromatic and xenobiotic compounds	Metatranscriptomic analysis of wheat rhizosphere deciphered taxonomic microbial communities and their multi-functionalities linked with degradation of aromatic and xenobiotic compounds.	Singh D. P. et al., 2018
12.	Activated-sludge microbiome.	Activated sludge	Heavy oil	*De novo* RNA seq strategy deciphered the high performance of nitrifers in degradation of heavy oil performance of reactor revealing unexpected linkage between carbon and nitrogen metabolisms in complex microbiomes.	Sato et al., 2019

Dual RNA-seq transcriptional profile is a better approach to understand the basic nature and mechanism of differently expressed genes in the host and symbiotic microbes at a time (Kaul et al., 2016). RNA seq allows for the detection of more differently expressed genes than a microarray alone does. Thus, recent advancements and developments in microarrays, RNA seq technology, transcriptomics, and metatranscriptomics revealed unexpected microbial diversity in aquatic and terrestrial environments with their synergistic relationships with humans, animals, plants, and other microorganisms (Perez-Losada et al., 2015; White et al., 2016; Moniruzzaman et al., 2017; Berg et al., 2018; Crump et al., 2018).

RNA seq technology is considered more efficient than traditional microarray platforms in gene expression profiling as it provides a wider quantitative range of expression level changes compared to microarrays (Roh et al., 2010; Shakya et al., 2019). The microarray technique requires a lot of effort and money to prepare custom-made microarrays. Furthermore, the target genes to be analyzed are limited in number and cannot cover the whole set of genes in the community. In contrast, a number of kits are now commercially available to carry out RNA-seq analysis, whereby a whole set of genes in the community can be quantitatively analyzed. Therefore, many studies are now performed by RNA-seq technology.

Lima-Morales et al. (2016) investigated the microbial organization and catabolic gene diversity of three types of contaminated soil under continuous long-term pollutant stress with benzene and benzene/toluene/ethylene/xylene (BTEX) to identify shifts in community structure and the prevalence of key genes for catabolic pathways. Moreover, *de novo* transcriptome synthesis gives new insights into and reveals basic information about non-model species without a genome reference. Hydrocarbon-degrading bacterium *Achromobacter* sp. was isolated from seawater, and indicated that the upregulation of enzymes such as dehydrogenases and monooxygenases, and novel genes associated with fatty acid metabolism is responsible for its enormous capability for hydrocarbon degradation and survival (Hong et al., 2016).

Metatranscriptomic analysis of the wheat rhizosphere identified dominant bacterial communities of diverse taxonomic phyla, including Acidobacteria, Cyanobacteria, Bacteroidetes, Steptophyta, Ascomycota, and Firmicutes, having functional roles in the degradation of various xenobiotic pollutants (Singh D. P. et al., 2018). Multiple enzymes such as isomerases, oxygenases, decarboxylases, reductases, kinases, and inner membrane translocators were identified that were associated with 21 different pathways for benzoates, aromatic amines, phenols, bisphenols, and other xenobiotics (Singh et al., 2016). An et al. (2020) elucidated the study of the transcriptome for the characterization of hexaconazole degrading strain *Sphingobacterium multivorum*, obtained from activated sludge. This strain was capable of degrading 85.6% hexaconazole in just 6 days and of generating three different metabolites, M1, M2, and M3, recognized as (2-(2,4-dichlorophenyl)-1-(1H-1,2,4-triazol-1-yl)hexane-2,5diol), (2-(2,4-dichlorophenyl) hexane-1,2-diol), and (1H-1,2,4-triazol), respectively. The results of transcriptome sequencing revealed the presence of 864 differential genes in which dehydrogenases, aldehydes, monooxygenases, and RND and AC transporters were upregulated. The M1 metabolite was perhaps generated due to the participation of monooxygenases.

Genomic and transcriptomic approaches were used by Sengupta et al. (2019) for gaining mechanistic insight into 4-nitrophenol (4-NP) degrading bacterium *Rhodococcus* sp. strain BUPNP1. This study identified a catabolic 43 gene cluster named *nph* that harbors not only mandatory genes for the breakdown of 4-NP into acetyl co-A and succinate by nitrocatechol, but also for other diverse aromatic compounds. An integrated approach of metagenomics and metatranscriptomics revealed the metabolic capabilities and synergistic relationship between *Sphingomonas* spp., *Pusillimonas* sp., and *Pseudomonas* sp. in the degradation of bisphenol A (BPA) (Yu K. et al., 2019).

Metatranscriptomic analysis of this interaction model demonstrated genes encoding the transcription of 1,2-bis(4-hydroxyphenyl)-2-propanol (1-BP) into 4-hydroxybenzaldehyde (4-HBD) and 4-hydroxy-acetophenone (4-HAP) via 3,4-dihydroxybenzoate (3,4-DHB) and 3-oxoadipate (3-ODP), respectively, to the tricarboxylic acid cycle (TCA)cycle. Marcelino et al. (2019) identified fungal species and subspecies in a mixed community by using metatranscriptomics. This study suggested a strain-level discrepancy between the *Cryptococcus* fungal species and their *in-situ* mock communities. Thus, transcriptomic analysis provides a large amount of gene information about the potential function of microbial communities in adaptation and survival in extreme environments (Singh D. P. et al., 2018; Mao et al., 2019; Marcelino et al., 2019).

### Proteomics

Proteins are crucial effectors of biological responses, stabler than RNAs in living organisms, and possibly confer an integral view of biological function expressed *in situ*; the term proteomics is put forward to study the entire set of proteins expressed in an organism (Ram et al., 2005; Singh, 2006; Hettich et al., 2013). Thus, proteomics has emerged as an interesting and fruitful technology to study protein expression (post-translational modifications, protein turnover, proteolysis, and changes in the corresponding gene expression) of the microbial world (Keller and Hettich, 2009; Aslam et al., 2017). Proteomics is a promising aspect of omic technologies in the field of microbiology, allowing for investigating the complete protein profile obtained in a straight line from a composite microbial population in a contaminated environment (Williams et al., 2013; Arsene-Ploetze et al., 2015; Wang et al., 2016). However, metaproteomics is used to decipher crucial information regarding the protein profiling of two diverse ecological units (Arsene-Ploetze et al., 2015). Proteomics has been used to identify microbial communities/microorganisms in various ecosystems including soil and sediment, activated sludge, marine and groundwater sediment, acid mine biofilms, and wastewater plants, as illustrated in Table 3 (Williams et al., 2013; Colatriano et al., 2015; Grob et al., 2015; Bastida et al., 2016; Jagadeesh et al., 2017). These studies revealed secret information related to protein synthesis, gene-expression stability, mRNA turnover, and protein–protein interaction networks in microbial communities in stress environments (Aslam et al., 2017).

**Table 3:** Microorganisms or microbial communities using proteomic and metaproteomic approaches in biodegradation.

**S. no**.	**Microorganisms**	**Xenobiotics/ pollutants**	**Technique used**	**Comments/result**	**References**
1.	*Microbacterium* Y2	Decarbodimethyl ether (BDE-209)	iTRAQ labeling and HRMS	Proteomic analysis showed that the over expression of haloacid dehalogenases, glutathione-S-transferases, and ATP-binding cassette (ABC) involve in the degradation of BDE-209 in *Microbacterium* Y2.	Yu Y. et al., 2019
2.	*Phanerochaete chrysosporium*	Terabromobisphenol A	High-performance liquid chromatography–mass spectrometry (HPLC–MS)	Proteome analysis showed upregulation of cyt P450 monooxygenase, glutathione-s-transferase, *O*-methyltransferase, and other oxidoreductases for the biotransformation of terabromobisphenol A via oxidative hydroxylation and reductive debromination.	Chen et al., 2019
3.	*Sphingobium chungbukense* DJ77	Polycyclic aromatic hydrocarbons (PAHs)	LC-MS	Proteomic analysis of PAH-degrading bacterial isolate predicted putative dehydrogenases, dioxygenases, and hydrolases involved in catabolic pathway of xenobiotic degradation.	Lee et al., 2016b
4.	*Burkholderia* sp. K24	Benzene, toluene, xylene, and aniline	LC-MS	Proteogenomic characterization of strain K24 revealed four independent degradation pathways for monocyclic aromatic hydrocarbons: benzene, toluene, xylene, and aniline.	Lee et al., 2016a
5.	*Acinetobacter* sp. KS-1	Benzoate	MALDI-TOF/MS	Proteome analysis of bacterium *Acinetobacter* sp. KS-1 showed two benzoate-degrading enzymes (catechol 1,2-dioxygenase, β-ketoadipate succinyl CoA transferase), and suggested that it degrades benzoate through β-ketoadipate pathway.	Kim et al., 2003
6.	*Acinetobacter radioresistens* S13	Aromatic compounds	Two-dimensional gel electrophoresis (2-DE) with *N*-terminal sequencing	Proteomic analysis revealed that six proteins were actively synthesized during aromatic-compound degradation, and enzymes of the β-ketoadipate pathway were observed and identified. DHB dehydrogenase, DBHDH, phenol hydroxylase oxygenase, PHO, catechol 1,2 dioxygenase, ketoadipyl-CoA thiolase, and muconolactone isomerase.	Mazzoli et al., 2007
7.	*Sphingomonas wittichii* RW1	Dioxins	MALDI-MS	Proteomic profiling of dioxin-degrading bacterium *Sphingomonas wittichii* RW1 identified a dioxin dioxygenase, *meta*-cleavage product hydrolase, and 2,3-dihydroxybiphenyl 1,2-dioxygenase proteins related to dioxin/dibenzofuran degradation.	Colquhoun et al., 2012
8.	*Sphingomonas haloaromaticamans* strain P3	*ortho*-phenylphenol	MALDI-TOF	Proteomic analysis of strain P3 explored the role of catabolic operons on the microbial bioremediation of *ortho*-phenylphenol (OPP). A total of 229 protein spots were identified that were differentially expressed in the presence of OPP. Among these, only 13 upregulated protein spots were associated with proteins having a putative role in OPP transformation.	Perruchon et al., 2017
9.	*Paecilomyces* sp. strain SF-8	PAHs	MALDI-TOF	Proteomic analysis revealed the expression of salicylaldehyde dehydrogenase, which is a key control protein of PAH degradation, especially overexpressed in strain SF-8.	Velmurgan et al., 2017
10.	*Mycobacterium* sp. strain 6PY1	Pyrene	2-DE using *N*-terminal sequencing	Proteomic analysis identified 40 induced pyrene-specific protein spots; nine proteins were detected. Two pyrene induced dioxygenases were differentially regulated.	Krivobok et al., 2003
10.	*Bacillus thuringiensis*	Cypermethrin	MALDI-TOF MS	Proteome analysis evaluated differential expressions of proteins during cypermethrin degradation.	Negi et al., 2016
11.	*Escherichia coli*	*p*-nitrophenol	MALDI-TOF/TOF-MS	Proteomic analysis showed differential expression of proteins during degradation of aromatic compounds such as *p*-nitrophenol.	Chakka et al., 2015
12.	Activated-sludge microbial community	PAHs	MALDI-TOF-MS	Metaproteomics analysis of PAH-treated sludge indicated the proteins derived from Burkholderiales population exhibiting differential protein expression profile and involved in PAH metabolism. The protein expression profile indicated that naphthalene was more liable to degradation than anthracene in sewage by Burkholderiales.	Li et al., 2019
13.	Microbial community	Toluene	Nano-liquid chromatography coupled with electrospray mass spectroscopy (Nano-LC-ESI-MS/MS)	Metaproteomic analysis of an anaerobic microbial community identified 202 unambiguous proteins derived from 236 unique spots. These proteins are involved in toluene degradation and mainly affiliated to the members of Desulfobulbaceae and several other Deltaproteobacteria.	Jehmlich et al., 2010

The inclusion of a proteomic approach helps to identify related enzymes and their metabolic pathways in the bioremediation of xenobiotics from various contaminated sites (Kim et al., 2004; Liu et al., 2017; Wei et al., 2017). Basically, there are four primary steps that involve proteomic analysis: (1) preparation of a biological sample; (2) extraction and separation of proteins by using two-dimensional gel electrophoresis (2D-GE); (3) protein gel images are examined by means of image-analysis software such as ImageMaster 2D or PDQuest; and (4) proteins are identified by using mass spectroscopy (MS)/MALDI-TOF/MS or LC-MS (Yates et al., 2009; Chakka et al., 2015; Velmurgan et al., 2017).

A combined protein profile of 20 PAH-induced proteins was studied by proteomics in *Mycobacterium vanbaalenii* PYR-1 grown in a PAH-supplemented culture medium (Kim et al., 2004). PAH exposure of five different compounds, i.e., pyrene, pyerene-4,5-quinone, phenanthrene, anthracene, and fluoranthere causes variation in protein composition, showing upregulation of multiple proteins for PAH treatment compared to an uninduced control sample. Several PAH-induced proteins were identified by LC-MS, including a catalase-peroxidase, a putative monooxygenase, a dioxygenase, a small subunit of naphthalene-inducible dioxygenase, and aldehyde dehydrogenase. The metaproteomic approach was employed by Bastida et al. (2016) to illustrate changes in metabolic activities during compost-treated bioremediation with the help of differential protein expressions in hydrocarbon-polluted soil. Metaproteomic analysis indicated that *Sphingomonadales* and uncultured bacteria are responsible for the degradation of hydrocarbons in compost-treated soil due to the higher expression of catabolic enzymes such as 2-hydroxymuconic semialdehyde, *cis*-dihydrodiol dehydrogenase, and catechol 2,3-dioxygenase, dioxygenases involved in the first oxygenation step of aromatic rings. Moreover, biphenyl-2,3-diol 1,2-dioxygenase, estradiol dioxygenase, and naphthalene 1,2-dioxygenase were identified in compost-treated samples. By using metaproteomics, this study explored the functional and phylogenetic relationship of contaminated soil, and the microbial key players involved in compost-assisted bioremediation.

Another study, undertaken by Vandera et al. (2015) demonstrated the comparative proteomic analysis of *Arthrobacter phenanivorans* Sphe3 on aromatic compounds phenanthrene and phthalates. The proteomic approach confirmed the involvement of several proteins in aromatic-substrate degradation by identifying those mediating the initial ring hydroxylation and ring cleavage of phenanthrene to phthalate. This study also revealed the presence of both the *ortho-* and the *meta-* cleavage pathway for the degradation of these aromatic compounds, and it also identified all proteins that take part in these pathways and are highly upregulated upon phthalate growth in comparison to phenanthrene growth.

Proteomic analysis of pyrene-degrading bacterium *Achromobacter xylosoxidans* PY4 was performed by Nzila et al. (2018), who identified a total of 1,094 proteins. Among these, 95 proteins were detected in glucose supplementation, and 612 proteins were detected in the presence of pyrene. Furthermore, 25 upregulated proteins were found to be involved in stress response and the progression of genetic information. Two upregulated proteins, 4-hydroxyphenylpyruvate dioxygenase and homogentisate 1,2-dioxygenase, are implicated in the lower degradation pathway of pyrene. Enzyme 4-hydroxyphenylpyruvate dioxygenase may catalyze the conversion of 2-hydroxybenzalpyruvic acid (metabolite of pyrene) to homogentisate. Homogentisate 1,2-dioxygenase is involved in the incorporation of 2 oxygen atoms to produce 4-maleyacetoacetate, which is an intermediate in several metabolic pathways.

Lee et al. (2016b)performed proteomic analysis of PAH-degrading bacterial isolate *Sphingobium chungbukense* DJ77. This strain exhibited outstanding degradation capability for various aromatic compounds. This study demonstrated the degradation of three xenobiotics compounds, i.e., phenanthrene, naphthalene, and biphenyls (PNB), and their associated proteins was analyzed by 2-DE and MALDI-TOF/MS analysis. During PNB biodegradation by bacterial cells, an alteration was observed in protein expression to cope with the stress condition. Comparative analysis of 2-DE results revealed that the intensity of 10 protein spots changes identically upon exposure to these xenobiotics in strain DJ77 (Lee et al., 2016b). Among these ten, five upregulated proteins with multiple functionalities were identified as putative dihydrodiol dehydrogenase (BphB), which catalyzes the NAD^+^-dependent oxidation of *trans-*dihydrodiols; 2,3-dihydrobisphenyl 1,2-dioxygenase (PhnQ), which cleaves the aromatic ring; and 2-hydroxy-6-oxo-6-phenylhexa-2,4-dienoate hydrolase (BphD), which degrades biphenyls and polychlorinated biphenyls. A part of the initial diverse catabolism of PNB by BphB, PhnQ, and BphD converged into the same catechol degradation branch. Now, catechol is first transformed into a ring-cleaved product, i.e., 2-hydroxymuconic semialdehyde by catechol 2,3-dioxygenase (PhnE). Moreover, it is assumed that this ring-cleaved product (2-hydroxymuconic semialdehyde) would be further degraded by 2-hydroxymuconic semialdehyde hydrolase (PhnD), and acetaldehyde dehydrogenase (PhnI) into a compound that can enter into the TCA cycle. Hence, these upregulated proteins, dehydrogenase, dioxygenase, and hydrolase, are involved in the catabolic degradation pathway of xenobiotics. The detection of intermediates from 2,3-dihydroxy-biphenyl degradation to pyruvate and acetyl-CoA by LC/MS analysis showed that ring-cleavage products entered the TCA cycle and were mineralized in strain DJ77. It was also suggested that strain DJ77 could completely degrade a wide range of PAHs via multiple catabolic pathways (Lee et al., 2016b).

The biodegradation mechanism of tetrabromobis-phenol A (TBBPA) was investigated in *Phanerochaete chrysosporium* by using a proteomic approach. iTRAQ quantitative analysis identified a total of 2,724 proteins in three biological samples. Compared to control TBBPA, stress caused 148 differentially expressed proteins in *P. chrysosporium*, among which 90 proteins were upregulated and 58 proteins were downregulated. The upregulation of cytochrome p450 monooxygenase, glutathione-*S*-transferase, *O*-methyltransferase, and other oxidoreductases is responsible for the biotransformation of TBBPA via oxidative hydroxylation and reductive debromination (Chen et al., 2019).

A biodegradation study of endocrine-disrupting compound 4-*n*-nonylphenol (4-*n***-**NP**)** by filamentous fungus *Metarhizium robertsii* was investigated by Szewczyk et al. (2014) with proteomic analysis. This suggested that the main biodegradation mechanism involves the consecutive oxidation of the alkyl chain and benzene, which consequently results in the complete decomposition of the 4-*n*-NP compound. Proteomic profiling explored the involvement of nitro-reductase-like proteins related to the oxidation–reduction and ROS defense systems, and mainly engaged group of proteins in the removal of 4-*n*-NP. Proteomic data obtained in this study could not clearly explain the mechanism of 4-*n*-NP biodegradation in the tested fungal strain, but allowed for the formulation of hypotheses that the over-expressed enzymes in the cultures with 4-*n*-NP could play a role in xenobiotic removal and the biodegradation process.

Bioremediation of decabromodiphenyl ether (BDE-209) was explored in *Microbacterium* Y2 in a polluted water-sediment system through proteomics (Yu Y. et al., 2019). Proteomic analysis showed that the overexpression of haloacid dehalogenases, glutathione s-transferase, and ATP-binding cassette (ABC) transporter might occupy important roles in BDE-209 biotransformation. Moreover, heat-shock proteins (HSPs), ribonuclease E, oligoribonuclease (Orn), and ribosomal proteins were activated to counter the BDE-209 toxicity. Thus, it is suggested that these proteins are implicated in microbial degradation, antioxidative stress, and glycolysis (Yu Y. et al., 2019).

### Metabolomics

Metabolomics is a well-established recent scientific technology, attributed toward the study of naturally occurring low-molecular weight (<1,000 Da) organic metabolites (organic acids; pyruvate, lactate, malate, formate, fatty acid-like acetate, etc.) inside a tissue, cell, or biofluid (Johnson et al., 2011; Malla et al., 2018; Withers et al., 2020). Metabolomics explores the relationships between organisms and the environment, such as organismal responses to abiotic stressors, including both natural factors such as temperature, and anthropogenic factors such as pollution, to investigate biotic–biotic interactions such as infections, and metabolic responses (Lindon et al., 2006; Griffiths, 2007; Mallick et al., 2019).

The combined use of metabolomics with these applications details the authentic collection of chemical outputs and inputs, which arbitrate the exchange of resources between the community and its host (Table 4; Theriot et al., 2014; Wang Y. F. et al., 2018). Metabolomic studies in environmental sciences have been directed toward understanding changes in the concentration of metabolites associated with exposing model organisms to toxic compounds, such as xenobiotics (Parisi et al., 2009; Seo et al., 2009).

**Table 4:** Microorganisms or microbial communities using metabolomic approaches in biodegradation.

**S. no**.	**Microorganisms/ microbial communities**	**Xenobiotics/ pollutants**	**Comments/results**	**References**
1.	*Burkholderia* sp. C3	*N-*methylcarbamates	Metabolomic analysis identified a total of 196 polar metabolites, 10 medium-to-long-chain fatty acids, and one type of macromolecule polyhydroxyalkanoates (PHA) in strain C3 in the degradation of *N*-methylcarbamate pesticides.	Seo et al., 2013
2.	*Lactobacillus plantarum*	Phorate	Metabolomic analysis identified a number of differential abundant metabolites in the presence of phorate by *L. plantarum*, and apparent alterations of metabolome profiles in cell-culture supernatant that contained phorate in comparison with the non-pesticide containing one.	Li et al., 2019
3.	*Mycobacterium* sp. DBP42 *Halomonas* sp. ATBC28	Plasticizers/phthalates (DBP/DHEP/ATBC)	Metabolomic study explored the metabolic potential of biofilm-producing marine bacterial isolates that colonize plastics, and revealed different mechanisms used for ester side chain removal from different plasticizers, i.e., DBP, DHEP, and ATBC.	Wright et al., 2020
4.	*Photobacterium ganghwense*	Cyfluthrin	Metabolomics explored the biotransformation pathway of cyfluthrin with the identification of 156 metabolites during biodegradation process.	Wang et al., 2019
5.	*Mycobacterium vanbaalenii* strain PYR-1	Benz[a]anthracene	Benz[a]anthracene was metabolized by *M. vanbaalenii* PYR-1 via four degradation pathways and produced numerous metabolites. Major metabolites identified by GC-MS and NMR spectral analysis were 3-hydrobenzo[f]isobenzofuran-1-one, 6-hydrofuran[3,4-g]chromene-2,8-dione, benzo[g]chromene-2-one, naphtha[2,1-g]chromen-10-one, 10-hydroxy-11methoxybenz[a]anthracene and 10, 11dimethoxybenz[a]anthracene.	Moody et al., 2005
6.	*Bacillus* sp. 3B6	Mesotrione	*Ex situ* NMR and LC-NMR techniques used to define the metabolic pathway involved during the biotransformation of mesotrione by *Bacillus* sp. 3B6. The complementarities of these NMR techniques identified two major metabolites (glutarate and MNBA), revealing the presence of a new metabolic pathway.	Durand et al., 2010
7.	*Drechslera* sp.	Methyl tertiary-butyl ether (MtBE)	Metabolomic analysis revealed the presence of two major bioactive metabolites, monocerin, and an alkyl substituted epoxycyclohexanone derivative that showed good antifungal activity and bioremediation.	d'Errico et al., 2020
8.	*Sinorhizobium* sp. C4	Phenanthrene	Comprehensive metabolite profiles, including polar metabolites, fatty acids, and polyhydroxyalkanoates were evaluated through untargeted metabolome analyses during phenanthrene degradation by *Sinorhizobium* sp. C4.	Keum et al., 2008
9.	*Bacillus thuringiensis* strain ZS-19	Cyhalothrin	Metabolomic analysis of strain ZS-19 through HPLC and GC-MS revealed biodegradation mechanisms of cyhalothrin.	Chen et al., 2015
11.	Soil microbial communities	Toxic pollutants	Metabolomics proposed to explore bioremediation potential, molecular changes, and metabolic pathways developed in microorganisms in contaminated environments.	Withers et al., 2020
12.	Soil microbial communities	Polycyclic aromatic hydrocarbons (PAHs)	Metabolomics with combined enzyme activity and sequencing analysis revealed the response of soil microbial communities to polycyclic aromatic hydrocarbon stress and their metabolic degradation pathway.	Li et al., 2018

Metabolomics approach was utilized to investigate the degradation mechanism of carbaryl and other *N*-methyl carbamates pesticides in *Burkholderia* sp. strain C3 (Seo et al., 2013). Metabolomes are dynamic and responsive to nutrient and environmental changes. The results of this study showed the metabolic adaptation of *Burkholderia* sp. C3 to carbaryl in comparison with glucose and nutrient broth. The metabolic changes were notably associated with the biosynthesis and metabolism of amino acids, sugars, PAH lipids, and cofactors. Differential metabolome analysis in response to different substrates identified 196 polar metabolites, 10 fatty acids, and 1 macromolecule (PHA) in this strain, and confirmed up-production metabolites in the pentose phosphate pathway, cysteine metabolism, amino acids, and disaccharides (Seo et al., 2013). Thus, this metabolomic study could provide detailed insights into bacterial adaptation to different metabolic networks, and the metabolism of toxic pesticides and chemicals.

Environmental pollutants cause alterations in microbial communities, which consequently changes biochemical and metabolic functions in soil microorganisms. Microbial degradation of cyfluthrin by *Photobacterium ganghwense* was investigated via a comparative metabolic approach (Wang et al., 2019). Metabolomics explored the biotransformation pathway of cyfluthrin with the identification of 156 metabolites during the biodegradation process. Recently, on the basis of interactions of indigenous soil microorganisms to PAH-contaminated soil, Li et al. (2018) elucidated that the majority of microbial metabolic functions were adversely affected to cope with PAH pollution. This study includes the combined study of enzyme activity and sequencing analysis with metabolomics, which further exposed the specific inhibition of soil metabolic pathways associated with carbohydrates, amino acids, and fatty acids due to microbial-community shifting under PAH stress.

Soil metabolomics is an effective approach to reveal the complex molecular networks and metabolic pathways operating in the soil microbial community. This approach can also be used to find biomarkers of soil contamination (Jones et al., 2014). High-throughput sequencing and soil metabolomics investigated the differential structures and functions of soil bacterial communities in the pepper rhizosphere and bulk soil under plastic greenhouse vegetable cultivation (PGVC) (Song et al., 2020). A total of 245 metabolites were identified, among which 11 differential metabolites were detected between rhizosphere and bulk soil, including organic acids and sugars that were positively and negatively correlated with the relative abundances of the differential bacteria. A starch and sucrose metabolic pathway was the most differentially expressed pathway in rhizospheric soil. The main functional genes participating in this pathway were predicted to be down regulated in rhizosphere soil. Sugar and organic acids as the main plant-root exudates in the rhizosphere, and they are also the main drivers of the shift in soil microbial community in the rhizosphere. These plant-root exudates act as an energy source to soil microbes, thus benefiting their growth (Kuzyakov and Blagodatskaya, 2015; Shi et al., 2015). Linear discriminant analysis (LDA) effect size (LEfSe) analysis showed that bacterial phyla of *Proteobacteria* and *Bacteroidetes* were significantly higher in rhizosphere soil, benefitting plant growth. Thus, the relationship between soil metabolites and microbial communities guides the regulation of plant rhizoprocesses through soil amendments to increase plant growth.

Durand et al. (2010) conducted metabolic analysis of *Bacillus* sp. to characterize the metabolic pathway for the biodegradation of mesotrione, a herbicide. Analysis was carried out by using LC-NMR and LC-MS, and the result of these instrumental analyses was the identification of six metabolites, of which the structures of four metabolites were suggested. Szewczyk et al. (2017) performed metabolic analysis of fungal strain *Metarhizium brunneum* ARSEF 2017 to predict a biodegradation-pathway metabolic background for the removal of ametryn, an *s*-triazene herbicide. Qualitative LC-MS/MS metabolomic analysis of ametryn biodegradation resulted in the generation of four metabolites, i.e., 2-hydroxy atrazine, ethyl hydroxylated ametryn, *S*-demethylated ametryn, and diethyl ametryn.

Wright et al. (2020) evaluated the metabolomic characterization of two potent marine bacterial isolates, *Mycobacterium* sp. DBP42 and *Halomonas* sp. ATBC28, capable of the degradation of phthalate and plasticizers such as ATBC, DBP, and DEHP. That study presented the molecular analysis of metabolites generated during biodegradation. A metabolomic study confirmed that DBP and ATBC were degraded through the sequential removal of ester side chains, and generated monobutyl phthalate and phthalate in the case of DBP degradation, and citrate in the case of ATBC degradation in *Mycobacterium* sp. However, DEHP degradation did not follow the same pathway as that observed for DBP and ATBC. It was suggested that DEHP degradation is initiated through hydroxylation of the ester side chain by monooxygenase, and may occur via the β-oxidation of fatty acid side chains directly on the DEHP molecule. Moreover, in comparison with *Mycobacterium* sp., *Halomonas* sp. did not confirm any detectable degradation intermediates for the degradation of plasticizers and phthalate, but it harbored an array of enzymes suggested to be responsible for the degradation of other aromatic compounds. Therefore, metabolomic analyses demonstrated changes that occur in the composition of metabolites, aiding to fully understand the shifts mechanisms of metabolites during the microbial degradation or mineralization of environmental pollutants (Lindon et al., 2006; Keum et al., 2008; d'Errico et al., 2020).

## Bioinformatics

Bioinformatic technology developed a new array of computational technologies that uses both information technology and biological sciences. This modern technology seeks information from multiple high-throughput biological techniques, and keeps all biological data, helping to investigate and decide the relationship among organic molecules, including macromolecular sequences, biochemical and metabolic pathways, protein expressions, metabolites, and structures (Fulekar and Geetha, 2008; Cooper et al., 2018; Dangi et al., 2018; Greene, 2018; Shekhar et al., 2020). Enormous amounts of data are generated from DNA, RNA, and protein sequences that need to be accurately executed; thus, bioinformatics has led to finding the best possible way to analyze such huge amounts of biological data via specific computational tools (Aora and Bar, 2014; Bhatt et al., 2021). Therefore, bioinformatic-associated tools are very important to understand the bioremediation of toxic pollutants. Bioinformatics provides superior information regarding the cellular, molecular, and genetical bases of xenobiotic degradation and detoxification (Kumar et al., 2016; Huang et al., 2020). There are a number of bioinformatic tools and applications that are available to use for biodegradation studies, as listed in Table 5.

**Table 5:** Bioinformatic databases and software tools used in biodegradation studies.

**Database/software tools**	**Features/functions**	**Web address/URL**	**References**
University of Minnesota Biocatalysis/ Biodegradation Database (UM-BBD)	Information about microbial catabolism and related biotransformation, and biodegradation pathways for xenobiotics and other hazardous pollutants.	https://umbbd.ethz.ch/	Ellis et al., 2006
Biodegradation Network-Molecular Biology (Bionemo)	Molecular knowledge about the structure and function of biodegradative genes and proteins.	https://bionemo.bioinfo.cnio.es	Carbajosa et al., 2009
Kyoto Encyclopedia of Genes and Genomes (KEGG)	Highly recommended for information regarding genetic, metabolic, enzymatic, and cellular progressions of microorganisms.	http://genome.ad.jp/kegg/	Kanehisa et al., 2017
National Center for Biotechnology Information (NCBI)	Public databases and software tools for storing, disseminating, and analyzing genome data.	http://www.ncbi.nih.gov/	Brown et al., 2015
OxDBase	Enzymatic database that contains all literature-cited information related to oxygenases.	www.imtech.res.in/raghava/oxdbase/	Arora et al., 2009
PathPred	Used to predict microbial-degradation pathways for pollutants.	http://genome.jp/tools.pathpred/	Moriya et al., 2010
MetaRouter	Maintains varied information regarding biodegradation networks, predicting biodegradative pathways for chemical compounds.	http://pdg.cnb.uam.es/MetaRouter	Pazos et al., 2005
MetaCyc	Provides information about enzymatic and metabolic mechanism/pathways.	http://metacyc.org	Capsi et al., 2016
Biocyc	Stores information related to organism-specific genome databases/pathways.	http://biocyc.org	Capsi et al., 2016
Molecular Evolutionary Genetic Analysis (MEGA 7.0)	Used for sequence alignment, hierarchical classification, and constructing phylogenetic tress.	www.megasoftware.net	Kumar et al., 2016
Biodegradative Strain Database (BSD)	Web-based database that provides detailed information about biodegradative bacteria and the hazardous chemicals that they degrade.	http://www.bsd.cme.msu.edu/	Urbance et al., 2003
KBase	Large-scale bioinformatic database that predicts and manages genomic data of plants and diverse microbial populations.	http://kbase.us/	Arkin et al., 2016
PAHbase	Functional PAH database that contains significant information on PAH-degrading bacteria, their occurrence phylogeny, metabolic pathways, and the genetic basis of their biodegradation capability.	http://www.pahbase.in	Kessner et al., 2008
ProteoWizard	Used for rapid proteomic analysis.	http://proteowizard.sourceforge.net/	Surani et al., 2011
BioRadBase	First comprehensive knowledge database that provides detailed information about the bioremediation of radioactive waste through microorganisms.	http://biorad.igib.res.in.	Reena et al., 2012
BiofOmics	Novel, systematic, and large-scale database for management and analysis of biofilm data from high-throughput experiment studies of microorganisms.	www.biofomics.org	Lourenco et al., 2012
Proteomics Identifications (PRIDE)	World's largest database for analysis of mass-spectrometry-based proteomic data. Includes generic standard-based format that can be annotated to capture data generated using any proteomic pipeline.	http://www.ebi.ac.uk/pride/	Vizcaino et al., 2016
MetaboLights	Database for metabolomic studies that provide primary research data and metadata for cross-platform and -species metabolomic studies.	http://www.ebi.ac.uk	Kale et al., 2016

MetaRouter is one of such application that is freely open and a modular architecture to a variety of consumers from any place in a safe and secure manner, just by connecting to an Internet server (Pazos et al., 2005). For the analysis of biodegradation studies, many bioinformatic resources are exclusively available. The University of Minnesota Biocatalysts/Biodegradation Database (UM-BBD) was introduced in 1995 and contains information regarding microbial catabolism and related biotransformation, biodegradation reactions, catabolic enzymes, and pathways for xenobiotics and other hazardous pollutants of various microorganisms. This database is connected to several other databases, such as BRENDA, ENZYME, ExPASy, and NCBI, to collect and store information related to gene structure and enzymes that take part in the biodegradation of environmental contaminants (Ellis et al., 2006). Genomic sequences of microorganisms with competent and efficient degradation abilities could be easily investigated via another widely used database, the National Center for Biotechnology Information (NCBI). It gives a detailed and complete pipeline for annotations, and comprehensive analysis of more than 6,000 microbial genomes (Brown et al., 2015). PRIDE (Vizcaino et al., 2016) is the world's largest data repository of mass-spectroscopy-based proteomic data, and MetaboLights (Kale et al., 2016) is a database for metabolomic experiments and derived information. The GenBank database is freely available in NCBI, and it provides most up-to-date and comprehensive DNA sequence information (Benson et al., 2013). Recently, there have been a number of databases such as CAMERA, MG-RAST, and IMG/M that were developed and employed for the analysis and in-depth understanding of diverse microbial populations, metabolic reconstruction, taxonomic affiliations, and their inter- and intra-relationship networks. Another database, Bionemo, developed by the structural computational-biology group at the Spanish National Cancer Research Center, gives information related to specific genes and proteins that take part in biodegradation reactions and metabolic pathways (Carbajosa et al., 2009). It provides insights into sequences, domains, protein structures, and regulatory elements, and transcription factors for their respective genes. Integrated bioinformatic approaches are employed for the metagenomic characterization of the soil microbial communities of different soil sites by using MetaPhlAn, KEGG, XLSX, and LEfSe bioinformatic databases to reveal the ancestral and functional characterization of diverse soil microbial populations (Arora et al., 2009; Xu et al., 2014; Kumar et al., 2016). The degradation or detoxification of xenobiotic pollutants through microbial communities is a highly considered and proficient remediation technology, and there is no single resource accessible that provides all the information with reference to environmental contaminants, microorganisms, and their bioremediation potentialities. Thus, these databases coalescing the detailed information about the nature of pollutants, their metabolic pathways, bioremediation microorganisms, catabolic genes, enzymes, and protein-expression profiles would be a significant tool to open up a new vista and enlighten future research science in the field of bioremediation.

## Conclusions

Microbial communities have great potential to mediate the successful biodegradation process of xenobiotic-contaminated soil/water environments. However, the greater part of mainstream microorganisms involved in bioremediation are still undefined because not all organisms in nature could be cultured under *in vitro* environments, but reside in viable-but-non-culturable (VBNC) environments. Thus, to explore the hidden knowledge of these VBNC organisms, recent advanced practices and sophisticated up-to-date technologies are highly desirable to understand the genetic and molecular biology of microorganisms. Newly developed molecular techniques offer promising approaches to address the in-depth characterization of microbial communities from molecule to gene. Recent omics technologies such as metagenomics, transcriptomics, and proteomics are helpful in obtaining information about nucleic acids, enzymes, catabolic genes, plasmids, and metabolic machineries and metabolites generated during the biodegradation process. However, the solitary employment of any individual omics technology is not sufficient to explore or illustrate secret information regarding microbial-remediation practices. Therefore, an interdisciplinary application of multiple omics studies highlights the perspectives of system biology for providing an integrative understanding between genes, proteins, and environmental factors responsible for the whole microbial-degradation process, and gives a new array of novel technologies, such as genome-editing and next-generation-sequencing tools CRISPR-Cas9, TALEN, and ZFNs, which are potent gene-editing tools that design microbes with specific degradation-function genes and provide unique insights into microbial remediation. Moreover, the successful execution of omics technologies could not be possible without the use of bioinformatic tools. The establishment of informative genomic and proteomic databases has been revolutionized by bioinformatics, which facilitates broad information about cellular- and metabolic-mechanism pathways for environmental pollutants. Hence, the involvement of these advanced technologies in the biological sciences shows the way to next-level research in the bioremediation potential of microorganisms, and exploits their capability to remove xenobiotic contamination.

## Data Availability Statement

The original contributions presented in the study are included in the article/supplementary material, further inquiries can be directed to the corresponding author/s.

## Author Contributions

Conceptualization and writing—original draft preparation: SM. Writing—review and editing: ZL, SP, WZ, PB, and SC. Supervision, funding acquisition, and project administration: SC. All authors contributed to the article and approved the submitted version.

## Conflict of Interest

The authors declare that the research was conducted in the absence of any commercial or financial relationships that could be construed as a potential conflict of interest.
